# Cardiac substructure dose in thoracic VMAT without explicit constraints: FMO versus SPO on uRT‐TPOIS with cross‐platform reference to eclipse PO

**DOI:** 10.1002/acm2.70911

**Published:** 2026-11-14

**Authors:** Chun Wu, Zhenmei Cao, Tingting Duan, Lixiang Han, Bin Wang, Weijie Lei

**Affiliations:** ^1^ Department of Radiation Oncology Xiamen Cancer Hospital Xiamen China; ^2^ Department of Radiation Oncology Second Affiliated Hospital of Xuzhou Medical University Xuzhou China; ^3^ Department of Radiation Oncology Xuzhou First People's Hospital Xuzhou China

**Keywords:** cardiac substructure, optimization algorithm, radiation‐induced cardiotoxicity, thoracic radiotherapy

## Abstract

**Background:**

Cardiac substructure doses are increasingly recognized as more specific predictors of radiation‐induced cardiac toxicity than whole‐heart metrics, yet these substructures are often left unconstrained in routine thoracic volumetric‐modulated arc therapy (VMAT) planning due to contouring burden or low prioritization.

**Purpose:**

This study aims to evaluate, in the absence of explicit cardiac substructure constraints, whether optimizer selection is associated with differences in cardiac substructure doses within a single treatment planning system (TPS), and to provide a descriptive cross‐system reference against a widely used clinical platform.

**Methods:**

Thirty patients (10 each across esophageal, breast, and non‐small cell lung cancers) initially treated with clinically delivered Photon Optimizer (PO) plans on the Eclipse platform were retrospectively replanned using two optimization algorithms on the uRT‐TPOIS platform: Fluence Map Optimization (FMO) and Stochastic Platform Optimization (SPO). No explicit dose constraints were applied to any cardiac substructure. Prespecified primary endpoints were mean heart dose (MHD), mean dose to the left anterior descending artery (LAD Dmean), mean dose to the left ventricle (LV Dmean), and mean lung dose (MLD), with the latter serving as a pulmonary toxicity reference. All other dosimetric parameters were analyzed as secondary/exploratory endpoints. Paired comparisons used the Wilcoxon signed‐rank test, with Benjamini–Hochberg false discovery rate correction applied separately within each cancer type and pairwise comparison for primary endpoints. Secondary endpoints were exploratory and not adjusted for multiplicity.

**Results:**

Intra‐platform comparison within uRT‐TPOIS showed that SPO was associated with lower MHD, LAD Dmean, and MLD than FMO in esophageal cancer, and lower MHD, LAD Dmean, and LV Dmean in breast cancer (*q* < 0.05). In NSCLC, no significant differences were observed for the primary endpoints. In secondary analyses, SPO was associated with shorter beam‐on time across all sites and improved target coverage in NSCLC. In descriptive cross‐system comparisons with Eclipse PO, SPO was associated with lower primary endpoint doses in esophageal and breast cancers, whereas in NSCLC, only MHD differed significantly, favoring PO. Exploratory analyses suggested additional differences in other cardiac substructures, but these were not adjusted for multiplicity and should be considered hypothesis‐generating.

**Conclusion:**

Under the specific uRT‐TPOIS protocol and without explicit cardiac substructure constraints, SPO was associated with lower primary endpoint doses than FMO in esophageal and breast cancers, but not in NSCLC. Cross‐system comparisons were descriptive and confounded by platform‐level factors. Whether explicit cardiac substructure constraints provide incremental benefit remains to be tested in dedicated within‐platform studies.

## 1 INTRODUCTION

Radiotherapy remains a cornerstone of curative management for thoracic malignancies (e.g., breast, esophageal, lung cancers).^1^, ^2^, ^3^ Despite significant survival gains from advanced systemic therapies, the therapeutic ratio is increasingly constrained by radiation‐induced toxicities. Minimizing radiation exposure to the heart and lungs is therefore critical due to the proximity of targets to these organs at risk (OARs).^4^, ^5^, ^6^, ^7^

Clinical evidence has established a dose‐dependent relationship between thoracic radiation exposure and cardiac toxicity as well as radiation pneumonitis (RP).^8^, ^9^, ^10^, ^11^, ^12^ Radiation‐induced cardiac toxicity encompasses both the pathophysiology of radiation‐induced heart disease (RIHD) and the clinically relevant endpoint of major adverse cardiac events (MACE). While MHD has traditionally served as the primary metric for cardiac dose assessment, growing evidence suggests its limited sensitivity in reflecting regional dose distribution patterns.^13^, ^14^ Recent studies have linked radiation dose to cardiac substructures, such as the left ventricle (LV) and coronary arteries (CA), with an increased risk of MACE and all‑cause mortality.^15^, ^16^, ^17^, ^18^ A recent meta‑analysis of 80 studies comprising 21 645 lung cancer patients further demonstrated that left anterior descending branch (LAD) V_15_≥10% was the only independent predictor of MACE, whereas MHD showed no significant association.^19^ These dose‐response relationships underscore the importance of managing doses to individual substructures, not merely whole‐heart metrics, to reduce long‐term cardiac risk.

To mitigate these risks, thoracic VMAT has been shown to achieve superior dose conformity while maintaining strict normal tissue constraints.^20^ However, the dosimetric quality of VMAT plans is influenced by both the treatment planning system (TPS) platform and its underlying optimization engine. The inherent modulation complexity of VMAT often results in increased dose heterogeneity within normal tissues, particularly manifesting as expanded volumes of low‐dose radiation exposure (e.g., lung V5).^21^, ^22^ Notably, lung V5 has emerged as a predictor of RP development, creating a complex dosimetric trade‐off between cardiac and pulmonary sparing in thoracic VMAT planning.^23^ Current clinical practice relies on multiple optimization approaches, including FMO and SPO on the uRT‑TPOIS platform, and the widely adopted PO on the Eclipse platform. FMO and SPO represent two different optimization algorithms within uRT‑TPOIS, whereas PO is the optimizer implemented in Eclipse.^24^, ^25^, ^26^ Each employs a distinct optimization strategy, which may lead to different dose fall‑off patterns and unintended exposure to unconstrained structures such as cardiac substructures.

Two clinically relevant questions arise from this context. First, do individual cardiac substructure constraints provide incremental dosimetric benefit over whole‐heart constraints alone? Second, in the absence of such substructure constraints, does optimization algorithm selection influence cardiac substructure doses, and is the magnitude of any difference dependent on the algorithm used? The first question has been partially addressed in prior literature,^16^, ^17^, ^18^ but its incremental benefit remains incompletely quantified. The second question, whether different optimizers implicitly produce different substructure doses despite meeting standard whole‐heart constraints, has received less attention and is the focus of the present study. Addressing this question is a prerequisite for future within‐platform studies comparing whole‐heart‐constrained and substructure‐constrained plans.

To address this gap, we compared FMO and SPO within the uRT‐TPOIS platform, where TPS, dose calculation engine, and iteration count were identical, thereby isolating algorithmic effects. In addition, we included clinically delivered PO plans on the Eclipse platform as a descriptive cross‐system reference, while explicitly acknowledging that these comparisons reflect composite system‐level factors, including TPS, dose engine, linac, and other system‐level factors, rather than optimizer performance alone. Our aims were: (1) to determine whether optimizer choice within the same TPS is associated with clinically meaningful differences in cardiac substructure doses under constraint‐free conditions; and (2) to provide a descriptive cross‐system reference using clinically delivered PO plans, interpreted as hypothesis‐generating.

## 2 MATERIAL AND METHODS

### 2.1 Patients

A total of 30 patients (10 patients per site) treated between October 2024 and October 2025 were analyzed, encompassing esophageal cancer, left‐sided breast cancer, and NSCLC. To maintain anatomical and dosimetric homogeneity, postoperative cases were excluded. The esophageal and NSCLC cohorts were restricted to mid‐to‐lower thoracic lesions treated with definitive radiotherapy. Specifically, the NSCLC group was selected for lesions immediately adjacent to the heart to evaluate high‐risk cardiac sparing. The breast cancer cohort was limited to patients with left‐sided lesions undergoing breast‐conserving therapy.

### 2.2 Sample Size Estimation

Due to the exploratory nature of this study and the need to include three distinct cancer types, esophageal, breast, and NSCLC, within a limited enrollment window, we enrolled 10 patients per site (total *N* = 30). Sensitivity power analysis using G*Power (Wilcoxon signed‐rank test for matched pairs, two‐tailed, α = 0.05, power = 0.80, sample size = 10 per cancer type) revealed a minimum detectable effect size of Cohen's *d* = 1.02. After Benjamini–Hochberg false discovery rate (FDR) correction for multiple primary endpoints, the effective minimum detectable effect size would be even larger. Therefore, nonsignificant findings should be interpreted as inconclusive rather than evidence of no difference.

### 2.3 CT simulation and contour delineations

CT simulation was performed on a Brilliance Big Bore CT scanner (Philips, Amsterdam, Netherlands) with patients immobilized in the supine position. For all patients in the cohorts, axial images were reconstructed at a 5 mm slice thickness for treatment planning.

All target volumes were initially delineated by a single radiation oncologist. The cardiac substructures, including LAD, left circumflex artery (LCX), right coronary artery (RCA), left main artery (LM), left atrium (LA), right atrium (RA), LV, and right ventricle (RV), were contoured using the AI‐based auto‐contouring tool AccuContour (Manteia),^27^ following the Feng atlas method.^28^ All contours were subsequently reviewed and validated by one senior expert radiation oncologist to ensure anatomical consistency and accuracy.

### 2.4 Treatment planning protocol

For each patient, the clinically delivered VMAT plan, optimized using the PO algorithm in Eclipse v13.6 on a VitalBeam accelerator, was adopted. Additionally, two alternative VMAT plans were generated for comparison using FMO and SPO in the uRT‐TPOIS vR001 system, which is integrated with a uRT‐linac 506c (United Imaging Healthcare, Shanghai, China) equipped with a 120‐leaf multileaf collimator (MLC). All plans were created by the same senior medical physicist applying identical standard clinical dose constraints (Table 1).

**TABLE 1:** Dose constraints for breast, esophageal, and NSCLC of PO/FMO/SPO plans.

	PO				FMO/SPO			
ROI	Type	Volume (%)	Dose (cGy)	Priority	Function	Description	Weight	Soft/Hard
Esophageal cancer								
PTV	Lower	99.9	5040	200	Min dose	Min dose 5040cGy	80	Hard
	Lower	97	5060	200	Min DVH	Min DVH 5060cGy to 97% Volume	100	Hard
	Upper	0	5200	180	Max dose	Max dose 5200 cGy	100	Hard
Lung‐PTV	Upper	50	500	150	Max DVH	Max DVH 500cGy to 50% Volume	20	Hard
	Upper	20	2000	150	Max DVH	Max DVH 2000cGy to 20% Volume	20	Hard
	Mean	/	900	120	Max EUD	Max EUD 900cGy a = 1	20	Hard
Heart	Upper	30	3000	150	Max DVH	Max DVH 3000cGy to 30% Volume	10	Hard
	Upper	20	4000	150	Max DVH	Max DVH 4000cGy to 20% Volume	10	Hard
	Mean	/	1900	150	Max EUD	Max EUD 1900cGy a = 1	20	Hard
Spinalcord	Upper	0	3800	180	Max dose	Max dose 3800 cGy	50	Hard
Spinalcord+3 mm	Upper	0	4000	150	Max dose	Max dose 4000 cGy	30	Hard
Body	Upper	0	5240	500	Max dose	Max dose 5240 cGy	500	Hard
R1	Upper	0	4840	160	Max dose	Max dose 4840 cGy	60	Hard
R2	Upper	0	4640	160	Max dose	Max dose 4640 cGy	60	Hard
Breast cancer								
PTV‐4005	Lower	99.9	4005	200	Min dose	Min dose 4005cGy	80	Hard
	Lower	97	4050	200	Min DVH	Min DVH 4050cGy to 97% Volume	100	Hard
PTV‐4800	Lower	99.9	4800	200	Min dose	Min dose 4800cGy	80	Hard
	Lower	97	4850	200	Min DVH	Min DVH 4850cGy to 97% Volume	100	Hard
	Upper	0	5200	180	Max dose	Max dose 5200 cGy	100	Hard
Lung‐L	Upper	26	500	150	Max DVH	Max DVH 500cGy to 26% Volume	20	Hard
	Upper	13	1600	150	Max DVH	Max DVH 1600cGy to 13% Volume	20	Hard
Lung‐R	Upper	3	500	80	Max DVH	Max DVH 500cGy to 3% Volume	10	Soft
Heart	Mean	/	150	80	Max EUD	Max EUD 150cGy a = 1	20	Hard
Spinalcord	Upper	0	500	80	Max dose	Max dose 500 cGy	20	Soft
Body	Upper	0	5500	500	Max dose	Max dose 5500 cGy	500	Hard
PTV‐ring	Upper	0	4205	180	Max dose	Max dose 4205 cGy	80	Hard
R4005‐1	Upper	0	3805	160	Max dose	Max dose 3805 cGy	60	Hard
R4005‐2	Upper	0	3605	160	Max dose	Max dose 3605 cGy	60	Hard
R4800‐1	Upper	0	4600	160	Max dose	Max dose 4600 cGy	60	Hard
R4800‐2	Upper	0	4400	160	Max dose	Max dose 4400 cGy	60	Hard
NSCLC								
PTV	Lower	99.9	6000	200	Min dose	Min dose 6000cGy	80	Hard
	Lower	97	6020	200	Min DVH	Min DVH 6020cGy to 97% Volume	100	Hard
	Upper	0	6200	180	Max dose	Max dose 6200 cGy	100	Hard
Lung‐PTV	Upper	50	500	150	Max DVH	Max DVH 500cGy to 50% Volume	20	Hard
	Upper	20	2000	150	Max DVH	Max DVH 2000cGy to 20% Volume	20	Hard
	mean	/	1000	150	Max EUD	Max EUD 1000cGy a = 1	20	Hard
Heart	Upper	30	3000	120	Max DVH	Max DVH 3000cGy to 30% Volume	10	Hard
	Upper	20	4000	120	Max DVH	Max DVH 4000cGy to 20% Volume	10	Hard
	mean	mean	1600	150	Max EUD	Max EUD 1600cGy a = 1	20	Hard
Spinalcord	Upper	0	3800	150	Max dose	Max dose 3800 cGy	50	Hard
Spinalcord+3 mm	Upper	0	4000	150	Max dose	Max dose 4000 cGy	30	Hard
Body	Upper	0	6200	500	Max dose	Max dose 6200 cGy	500	Hard
R1	Upper	0	5800	160	Max dose	Max dose 5800 cGy	60	Hard
R2	Upper	0	5600	160	Max dose	Max dose 5600 cGy	60	Hard

*Note*: Prescription doses: esophageal 50.4 Gy; breast 40.05 Gy/15 fx; NSCLC 60 Gy. Constraints followed institutional protocols based on NCCN/ESTRO guidelines. R1 / R4005‐1 / R4800‐1: ring between 0.5‐1 cm external expansions of PTV / PTV‐4005 / PTV‐4800, respectively. R2 / R4005‐2 / R4800‐2: ring between 1‐1.5 cm external expansions of PTV / PTV‐4005 / PTV‐4800, respectively.

Abbreviations: DVH, Dose‐Volume Histogram; EUD, Equivalent Uniform Dose; FMO, Fluence Map Optimization; PO, Photon Optimizer; SPO, Stochastic Platform Optimization.

Esophageal and NSCLC plans employed two coplanar full arcs (clockwise/counter‐clockwise) with collimator angles of 10° and 350°, respectively. In contrast, breast cancer plans utilized a two‐partial‐arc technique to optimize contralateral breast sparing. The arcs spanned 300° to 150° (counter‐clockwise), with a gantry angle gap (350° to 100°) to avoid direct beam entry, and the collimator angles were set at 10° (clockwise) and 350° (counter‐clockwise).

All plans were calculated using 6 MV X‐rays at 600 MU/min with a 2.5 mm dose grid resolution. Automatic jaw tracking was enabled across all algorithms. Dose calculations were performed using each system's native algorithm: Convolution for FMO and SPO plans within uRT‐TPOIS, and the Anisotropic Analytical Algorithm (AAA) for PO plans within Eclipse.

### 2.5 Iteration Protocol and Plan Comparability

PO plans were originally optimized with 100–200 manual iterations in Eclipse, whereas FMO and SPO plans were generated in uRT‐TPOIS with a fixed iteration setting. In uRT‐TPOIS, 50 iterations are commonly used for thoracic cases, although institutional protocols may vary (e.g., some breast cancer protocols use 30 iterations); therefore, 50 iterations were adopted uniformly for FMO and SPO in this study. This difference in iteration protocol represents a confounder in cross‐platform comparisons. The adequacy of 50 iterations was evaluated in a DVH convergence analysis (Tables S1A, S1B, and Figure S1).

Dose normalization was performed by scaling the prescription dose normalization factor so that 95% of the PTV volume was covered by the 100% prescription isodose across all plans. Because this procedure uniformly scales all doses, OAR doses may increase proportionally, and some OAR constraints that were marginally met before normalization may be exceeded after normalization. Any changes in OAR compliance after normalization are documented in Tables S2 (provided as Excel spreadsheets). All plans were compared under the same normalization procedure, preserving the internal validity of the intra‐platform comparison.

### 2.6 Plan evaluation

Plan quality was quantitatively assessed through DVH analysis. For target volumes, the following metrics were compared: D_99_ (the dose covering 99% of the volume), overdose parameters, conformity index (CI), homogeneity index (HI). CI and HI were calculated using:

(1) $$\mathrm{CI}=\frac{{\mathrm{PTV}}_{\mathrm{ref}}}{V_{\mathrm{PTV}}}\times \frac{{\mathrm{PTV}}_{\mathrm{ref}}}{V_{\mathrm{ref}}}$$

where PTV_ref_ = target volume receiving prescription dose, V_PTV _= total target volume, V_ref _= total volume receiving the prescription dose.

(2) $$\mathrm{HI}=\frac{D_{2}-D_{98}}{D_{50}}\;$$

where D_2_, D_50_ and D_98_ correspond to radiation doses delivered to 2%, 50%, and 98% of the PTV, respectively. Optimal plan quality is indicated by CI values approaching 1, with lower HI values preferred.

Delivery efficiency was evaluated based on the total number of monitor units (MUs) and beam‐on time. Beam‐on time was defined as the duration from the initiation of the first irradiation field to the completion of the final field, encompassing gantry rotation and MLC motion.

### 2.7 Dose verification

Delivery accuracy was verified using portal dosimetry for PO plans and the Delta4 phantom for FMO/SPO plans. The gamma pass rate (GPR) was calculated by comparing TPS‐calculated doses with measured doses, applying global gamma criteria of 3%/3 mm and 2%/2 mm with a 10% dose threshold (i.e., excluding regions receiving < 10% of the prescription dose from gamma analysis). Given system compatibility constraints, different verification phantoms were used: portal dosimetry for PO plans and Delta4 for FMO/SPO plans. The primary purpose was to confirm deliverability rather than to compare absolute dosimetric accuracy across platforms. Therefore, GPR results are reported as a quality assurance benchmark rather than a comparative metric of platform performance.

### 2.8 Statistical analysis

Quantitative data are presented as mean ± standard deviation (SD). Statistical analysis was performed using SPSS software (version 26.0; IBM Corp., Armonk, NY, USA). Paired comparisons were performed using the Wilcoxon signed‐rank test (two‐sided) for all endpoints. Paired differences are reported as Hodges–Lehmann estimates with 95% confidence intervals. Multiple comparisons among primary endpoints (MHD, LAD Dmean, LV Dmean, and MLD) were controlled using the Benjamini–Hochberg FDR procedure, applied separately within each cancer type and each pairwise comparison (FMO vs. SPO; SPO vs. PO), and adjusted *q*‐values are reported. For breast cancer, mean dose to the left lung was used as the pulmonary reference instead of MLD, as only the ipsilateral lung is clinically relevant. Secondary and exploratory endpoints are reported with raw *p*‐values and should be interpreted as hypothesis‐generating. For all analyses, a two‐sided *p*‐value < 0.05 was considered statistically significant, with the understanding that *p*‐values for inter‐platform comparisons are descriptive rather than confirmatory due to the confounding factors noted above. Effect sizes (Cohen's d for paired samples) were calculated as the mean paired difference divided by the standard deviation of the differences, and interpreted as: small (d < 0.5), medium (0.5 ≤ d < 0.8), and large (d ≥ 0.8). Effect sizes should be interpreted with caution given the limited sample size and the exploratory nature of the study (see Section 2.2).

## 3 RESULTS

### 3.1 Intra‑platform Algorithmic Differences: FMO versus SPO on uRT‑TPOIS

#### 3.1.1 Esophageal Cancer

Among the prespecified primary endpoints (Table 2), SPO was associated with lower MHD (−0.86 Gy, 95% CI [−1.72, −0.38]; *q* = 0.010; *d* = 1.15), MLD (−0.23 Gy, 95% CI [−0.46, −0.12]; *q* = 0.010; *d* = 1.13), and LAD Dmean (−2.92 Gy, 95% CI [−6.12, −0.65]; *q* = 0.029; *d* = 0.88) compared with FMO. LV Dmean did not differ significantly (−1.32 Gy, 95% CI [−3.18, 0.92]; *q* = 0.14; *d* = 0.49). In exploratory analyses (Table S3), SPO was associated with lower lung V5 (−3.48%, 95% CI [−5.68, −0.86]; *p* = 0.009; *d* = 1.10), LAD V10 (−20.17%, 95% CI [−38.69, −2.37]; *p* = 0.022; *d* = 0.80), RV V10 (−12.29%, 95% CI [−19.87, −4.86]; *p* = 0.007; *d* = 1.17), and LCX V10 (−6.53%, 95% CI [−12.46, −2.31]; *p* = 0.012; *d* = 1.03). SPO also required fewer MUs (−216.3, 95% CI [−293.5, −148.4]; *p* = 0.005; *d* = 2.48) and shorter beam‐on time (−16.8 s, 95% CI [−27.1, −8.7]; *p* = 0.007; *d* = 1.37). Target coverage (D99, HI, CI) was comparable between algorithms.

**TABLE 2:** Statistical details for prespecified primary endpoints in paired comparisons.

Cancer type	Comparison	Endpoint	FDR q	Paired difference (95% CI) (Gy)	Cohen's d
Esophageal cancer					
	FMO vs. SPO	MHD	0.01	−0.86(−1.72, −0.38)	1.15
	FMO vs. SPO	MLD	0.01	−0.23(−0.46, −0.12)	1.13
	FMO vs. SPO	LAD Dmean	0.029	−2.92(−6.12, −0.65)	0.88
	FMO vs. SPO	LV Dmean	0.14	−1.32(−3.18, 0.92)	0.49
	PO vs. SPO	MHD	0.018	−1.41(−2.22, −0.43)	1.19
	PO vs. SPO	MLD	0.037	−0.38(−0.69, −0.06)	0.89
	PO vs. SPO	LAD Dmean	0.018	−3.1(−4.24, −1.98)	2.05
	PO vs. SPO	LV Dmean	0.037	−2.28(−4.0, −0.63)	0.89
Breast cancer					
	FMO vs. SPO	MHD	0.0493	−12.7(−23.0, −2.7)	0.95
	FMO vs. SPO	Left Lung Dmean	0.074	−0.27(−0.56, 0.01)	0.6
	FMO vs. SPO	LAD Dmean	0.028	−1.72(−3.54, −0.84)	1.08
	FMO vs. SPO	LV Dmean	0.0493	−0.23(−0.41, −0.03)	0.92
	PO vs. SPO	MHD	0.007	−1.7(−2.2, −1.0)	2.14
	PO vs. SPO	Left Lung Dmean	0.022	−1.07(−1.8, −0.24)	1.02
	PO vs. SPO	LAD Dmean	0.007	−8.18(−11.02, −5.29)	2.41
	PO vs. SPO	LV Dmean	0.007	−2.45(−3.37, −1.97)	2.78
NSCLC					
	FMO vs. SPO	MHD	0.186	−0.47(−1.12, 0.14)	0.57
	FMO vs. SPO	MLD	0.148	−0.24(−1.47, −0.02)	0.5
	FMO vs. SPO	LAD Dmean	0.677	−0.23(−1.57, 2.11)	0.05
	FMO vs. SPO	LV Dmean	0.799	0.11(−0.73, 1.06)	0.11
	PO vs. SPO	MHD	0.02	2.01(0.71, 3.11)	1.24
	PO vs. SPO	MLD	0.185	−0.33(−1.36, 0.19)	0.47
	PO vs. SPO	LAD Dmean	0.241	0.42(−0.19, 2.68)	0.44
	PO vs. SPO	LV Dmean	0.185	0.46(−0.07, 1.91)	0.48

*Note*: Paired comparisons are performed using the Wilcoxon signed‐rank test (two‐sided) (*n* = 10 patients per cancer type). Paired differences are reported as Hodges‐Lehmann estimates with 95% confidence intervals. Benjamini–Hochberg FDR‐adjusted q‐values are reported.

Abbreviations: FMO, Fluence Map Optimization; LAD Dmean, mean dose to the left anterior descending artery; LV Dmean, mean dose to the left ventricle; MHD, mean heart dose; MLD, mean lung dose; PO, Photon Optimizer; SPO, Stochastic Platform Optimization.

#### 3.1.2 Breast Cancer

Among the prespecified primary endpoints (Table 2), SPO was associated with lower MHD (−0.13 Gy, 95% CI [−0.23, −0.03]; *q* = 0.049; *d* = 0.95), LAD Dmean (−1.72 Gy, 95% CI [−3.54, −0.84]; *q* = 0.028; *d* = 1.08), and LV Dmean (−0.23 Gy, 95% CI [−0.41, −0.03]; *q* = 0.049; *d* = 0.92) compared with FMO. Left lung Dmean did not differ significantly (−0.27 Gy, 95% CI [−0.56, 0.01]; *q* = 0.074; *d* = 0.60). In exploratory analyses (Table S3), SPO was associated with higher hotspot volumes (V117: 3.19%, 95% CI [1.36, 6.12]; *p* = 0.007; *d* = 1.10; V120: 2.90%, 95% CI [0.88, 6.37]; *p* = 0.013; *d* = 0.91). SPO was associated with lower LAD V30 (−8.14%, 95% CI [−14.75, −1.82]; *p* = 0.008; *d* = 0.92) and LV V30 (−0.35%, 95% CI [−0.75, −0.04]; *p* = 0.038; *d* = 0.70). SPO required fewer MUs (−74.2, 95% CI [−137.8, −16.8]; *p* = 0.017; *d* = 1.01) and shorter beam‐on time (−40.0 s, 95% CI [−46.1, −35.3]; *p* = 0.005; *d* = 5.37). Target coverage (D99, HI, CI) was comparable between algorithms.

#### 3.1.3 NSCLC

Among the prespecified primary endpoints (Table 2), no significant differences were observed between SPO and FMO after FDR correction: MHD (−0.47 Gy, 95% CI [−1.12, 0.14]; *q* = 0.186; *d* = 0.57), MLD (−0.24 Gy, 95% CI [−1.47, −0.02]; *q* = 0.148; *d* = 0.50), LAD Dmean (−0.23 Gy, 95% CI [−1.57, 2.11]; *q* = 0.677; *d* = 0.05), and LV Dmean (+0.11 Gy, 95% CI [−0.73, 1.06]; *q* = 0.799; *d* = 0.11). In exploratory analyses (Table S3), target coverage parameters favored SPO with higher D99 (+1.03 Gy, 95% CI [0.60, 1.48]; *p* = 0.005; *d* = 1.12), lower HI (−0.031, 95% CI [−0.043, −0.020]; *p* = 0.005; *d* = 1.73), better CI (+0.035, 95% CI [0.015, 0.070]; *p* = 0.011; *d* = 1.04), lower V105 (−17.35%, 95% CI [−36.41, −5.58]; *p* = 0.005; *d* = 0.95), and lower V107 (−23.90%, 95% CI [−36.71, −14.50]; *p* = 0.007; *d* = 1.51). SPO also required fewer MUs (−255.1, 95% CI [−286.2, −180.2]; *p* = 0.005; *d* = 3.18) and shorter beam‐on time (−19.4 s, 95% CI [−24.4, −10.7]; *p* = 0.013; *d* = 1.59).

### 3.2 Inter‑platform system‑level differences: uRT‑TPOIS versus eclipse

In this descriptive cross‐system comparison, SPO and PO refer to plans generated on uRT‐TPOIS and Eclipse, respectively.

#### 3.2.1 Esophageal cancer

Among the prespecified primary endpoints (Table 2), SPO was associated with lower MHD (−1.41 Gy, 95% CI [−2.22, −0.43]; *q* = 0.018; *d* = 1.19), MLD (−0.38 Gy, 95% CI [−0.69, −0.06]; *q* = 0.037; *d* = 0.89), LAD Dmean (−3.10 Gy, 95% CI [−4.24, −1.98]; *q* = 0.018; *d* = 2.05), and LV Dmean (−2.28 Gy, 95% CI [−4.00, −0.63]; *q* = 0.037; *d* = 0.89) compared with PO.

In exploratory analyses (Table S3), SPO was associated with higher D99 (+0.67 Gy, 95% CI [0.10, 1.09]; *p* = 0.017; *d* = 0.96) and lower HI (−0.011, 95% CI [−0.022, −0.001]; *p* = 0.065; *d* = 0.55), while CI, V105, and V107 were comparable. SPO was associated with lower lung V5 (−11.9%, 95% CI [−13.53, −9.13]; *p* = 0.005; *d* = 3.46) and lower doses to several cardiac substructures, including LAD V5 (−35.5%, 95% CI [−65.37, −0.80]; *p* = 0.021; *d* = 0.98), LAD V10 (−17.6%, 95% CI [−29.85, −8.13]; *p* = 0.011; *d* = 1.20), LA V20 (−5.1%, 95% CI [−8.22, −2.23]; *p* = 0.005; *d* = 1.30), LV V5 (−13.1%, 95% CI [−37.93, −3.55]; *p* = 0.009; *d* = 0.77), LV V10 (−16.7%, 95% CI [−32.63, −8.32]; *p* = 0.005; *d* = 1.17), LV V20 (−3.7%, 95% CI [−6.73, −1.53]; *p* = 0.013; *d* = 1.07), RV Dmean (−1.21 Gy, 95% CI [−2.60, −0.30]; *p* = 0.028; *d* = 0.78), and LCX Dmax (−1.15 Gy, 95% CI [−1.97, −0.33]; *p* = 0.009; *d* = 1.14). SPO also required fewer MUs (−99.7, 95% CI [−148.8, −66.4]; *p* = 0.007; *d* = 1.62) and shorter beam‐on time (−7.9 s, 95% CI [−11.1, −5.1]; *p* = 0.005; *d* = 2.20).

#### 3.2.2 Breast cancer

Among the prespecified primary endpoints (Table 2), SPO was associated with lower MHD (−1.7 Gy, 95% CI [−2.2, −1.0]; *q* = 0.007; *d* = 2.14), lower mean dose to the left lung (−1.07 Gy, 95% CI [−1.80, −0.24]; *q* = 0.022; *d* = 1.02), lower LAD Dmean (−8.18 Gy, 95% CI [‐11.02, ‐5.29]; *q* = 0.007; *d* = 2.41), and lower LV Dmean (−2.45 Gy, 95% CI [−3.37, −1.97]; *q* = 0.007; *d* = 2.78) compared with PO.

In exploratory analyses (Table S3), SPO was associated with higher PTV hotspot volumes (V117: +2.98%, 95% CI [0.28, 6.82]; *p* = 0.020; *d* = 0.79; V120: +2.50%, 95% CI [0.44, 5.02]; *p* = 0.020; *d* = 0.91). SPO was associated with lower Heart V10 (−5.1%, 95% CI [−6.7, −3.6]; *p* = 0.005; *d* = 2.23), left lung V5 (−8.4%, 95% CI [−12.2, −4.2]; *p* = 0.007; *d* = 1.50), LAD V5 (−10.77%, 95% CI [−26.47, −0.38]; *p* = 0.018; *d* = 0.83), LAD V10 (−16.04%, 95% CI [−35.77, −5.96]; *p* = 0.012; *d* = 0.93), LAD V20 (−23.93%, 95% CI [−36.89, −13.25]; *p* = 0.005; *d* = 1.53), LAD V30 (−29.16%, 95% CI [−42.08, −14.92]; *p* = 0.005; *d* = 1.54), LV V5 (−13.16%, 95% CI [−18.58, −9.07]; *p* = 0.005; *d* = 2.21), LV V10 (−7.32%, 95% CI [−11.31, −5.01]; *p* = 0.005; *d* = 1.75), LV V20 (−4.15%, 95% CI [−5.69, −2.90]; *p* = 0.005; *d* = 2.29), LV V30 (−1.60%, 95% CI [−2.96, −0.50]; *p* = 0.022; *d* = 0.82), RV Dmean (−1.49 Gy, 95% CI [−1.93, −1.24]; *p* = 0.005; *d* = 3.29), RV V5 (−10.18%, 95% CI [−13.40, −6.82]; *p* = 0.005; *d* = 2.52), LA Dmean (−0.42 Gy, 95% CI [−0.62, −0.33]; *p* = 0.005; *d* = 2.24), and RA Dmean (−0.28 Gy, 95% CI [−0.59, −0.16]; *p* = 0.005; *d* = 1.18). SPO required numerically more MUs (+79.3, 95% CI [‐30.9,192.5]; *p* = 0.139; *d* = 0.54) and longer delivery time (+7.1 s, 95% CI [1.1, 10.0]; *p* = 0.047; *d* = 1.05).

#### 3.2.3 Non‑small cell lung cancer

Among the prespecified primary endpoints (Table 2), only MHD differed significantly after FDR correction, with SPO associated with higher MHD than PO (+2.01 Gy, 95% CI [0.71, 3.11]; *q* = 0.020; *d* = 1.24). No significant differences were observed for MLD (−0.33 Gy, 95% CI [−1.36, 0.19]; *q* = 0.185; *d* = 0.47), LAD Dmean (+0.42 Gy, 95% CI [−0.19, 2.68]; *q* = 0.241; *d* = 0.98), or LV Dmean (+0.46 Gy, 95% CI [−0.07, 1.91]; *q* = 0.185; *d* = 0.48).

In exploratory analyses (Table S3), SPO was associated with higher D99 (+1.35 Gy, 95% CI [0.88, 1.94]; *p* = 0.005; *d* = 1.87) and lower lung V5 (−9.75%, 95% CI [−12.92, −4.63]; *p* = 0.007; *d* = 1.62), but numerically higher V107 (+17.90%, 95% CI [−1.29, 32.68]; *p* = 0.074; *d* = 0.80), lower CI (−0.021, 95% CI [−0.05, −0.01]; *p* = 0.005; *d* = 0.95), and higher doses to several cardiac substructures, including LAD V5 (+6.78%, 95% CI [2.40, 14.73]; *p* = 0.009; *d* = 0.98), LV V5 (+3.16%, 95% CI [1.57, 7.13]; *p* = 0.017; *d* = 0.95), LA V30 (+5.68%, 95% CI [3.38, 8.44]; *p* = 0.005; *d* = 1.59), LA Dmean (+2.59 Gy, 95% CI [1.28, 4.19]; *p* = 0.007; *d* = 1.37), and RV Dmean (+1.66 Gy, 95% CI [0.36, 4.13]; *p* = 0.013; *d* = 0.83). SPO also required fewer MUs (−137.3, 95% CI [−185.3, −93.0]; *p* = 0.005; *d* = 2.31) and shorter beam‐on time (−3.0 s, 95% CI [−3.8, −2.4]; *p* = 0.005; *d* = 3.13).

### 3.3 Dose verification

All plans achieved mean GPRs above the clinically acceptable threshold of 95% at both criteria (Table 3). At 3%/3 mm, GPR ranged from 96.43% to 99.54% (uRT‐TPOIS: 98.48%–99.54%; PO: 96.43%–97.68%); at 2%/2 mm, GPR ranged from 95.03% to 99.05% (uRT‐TPOIS: 97.71%–99.05%; PO: 95.03%–95.51%), confirming deliverability of all planning strategies.

**TABLE 3:** Gamma pass rates for the three planning strategies in thoracic tumors.

	FMO	SPO	PO
Esophagus cancer			
GPR (3%,3 mm)	98.8 ± 0.68	98.93 ± 0.66	97.68 ± 1.22
GPR (2%,2 mm)	97.71 ± 1.11	97.78 ± 1.09	95.31 ± 2.57
Breast cancer			
GPR (3%,3 mm)	99.54 ± 0.51	99.45 ± 0.61	96.43 ± 1.78
GPR (2%,2 mm)	99.05 ± 0.76	99.05 ± 0.87	95.51 ± 1.64
NSCLC			
GPR (3%,3 mm)	98.57 ± 1.03	98.48 ± 1.06	97.45 ± 2.09
GPR (2%,2 mm)	97.98 ± 0.72	98.02 ± 0.64	95.03 ± 2.28

*Note*: Gamma pass rates were obtained using different verification phantoms (portal dosimetry for PO; Delta4 for FMO/SPO) and are reported as a quality assurance benchmark, not as a direct cross‐platform comparison of delivery accuracy. Values are expressed as mean ± SD.

Abbreviations: FMO, Fluence Map Optimization; GPR, Gamma Pass Rate; PO, Photon Optimizer; SPO, Stochastic Platform Optimization.

## 4 DISCUSSION

Under the specific uRT‐TPOIS protocol studied, we examined whether cardiac substructure dosimetry differed according to optimizer selection in the absence of explicit substructure constraints, using Eclipse PO plans as a descriptive cross‐system reference. The principal finding was that optimizer selection was associated with cancer‐specific differences in cardiac substructure doses within the uRT‐TPOIS platform. In contrast, cross‐system differences were confounded by multiple platform‐level factors and therefore cannot be attributed to the optimization algorithm alone. These exploratory findings provide a rationale for future within‐platform studies comparing whole‐heart‐constrained and substructure‐constrained plans, rather than for algorithm or platform switching.

Several possible mechanisms may underlie these observations, although they remain speculative. PO, FMO, and SPO employ distinct optimization strategies,^24^, ^25^, ^26^ which may lead to differences in dose fall‐off patterns and OAR sparing. For example, SPO has been described as using a stochastic optimization framework,^24^ which may contribute to the observed differences in low‐dose bath regions (e.g., lung V5 in esophageal cancer and NSCLC); however, the specific mechanism was not directly tested in this study. Within the uRT‐TPOIS platform, where TPS, dose engine, and iteration count were identical, the observed differences between SPO and FMO are unlikely to be explained by these platform‐level factors, although dedicated mechanistic studies are needed to confirm the underlying algorithmic causes.

In the descriptive cross‐system comparison, esophageal and breast cancer plans generated with SPO showed concurrent reductions in lung V5 and cardiac substructure doses relative to PO plans, whereas in NSCLC, SPO was associated with lower lung V5 but higher MHD. We hypothesize that this NSCLC‐specific pattern may be related to extensive PTV‐heart overlap in this selected cohort. In an exploratory post hoc analysis, no significant correlation was found between overlap volume and MHD (*n* = 10, underpowered), suggesting that other factors may also contribute. This pattern warrants further investigation with dose distribution analysis and larger cohorts.

A key methodological observation is that whole‐heart metrics such as MHD may not fully capture substructure‐specific differences, as the magnitude, statistical significance, and even direction of changes in MHD and substructure doses (e.g., LAD and LV) were not always concordant across cancer types and platforms. This is relevant given established dose‐response relationships linking LAD dose to MACE^29^ and LV dose to heart failure.^30^ However, our study was not designed to quantify the incremental benefit of explicitly constraining these substructures over whole‐heart constraints alone. We fully agree that the ideal study design would first compare whole‐heart‐constrained and substructure‐constrained plans within each algorithm, and then assess whether the magnitude of benefit is algorithm‐dependent. Such a sequential design is a critical next step. The present study provides the rationale for that step by demonstrating that unconstrained substructure doses vary meaningfully by optimizer under identical platform conditions. When feasible, directly constraining cardiac substructures may be a preferred strategy, but its incremental benefit remains to be quantified in dedicated within‐platform studies.

Mean GPR exceeded 95% at both 3%/3 mm and 2%/2 mm criteria for all plans, confirming deliverability. Because different verification phantoms were used, GPR differences should not be interpreted as a comparative measure of delivery accuracy.

Several limitations should be acknowledged. First, the cross‐platform comparisons (uRT‐TPOIS vs. Eclipse) are confounded by multiple system‐level differences, including TPS platform, dose calculation engine (convolution vs. AAA), linac, MLC, optimization workflow, planner involvement, and dose evaluation. These differences preclude causal attribution of the observed cross‐platform findings to the optimizer alone, and these comparisons should therefore be interpreted strictly as descriptive system‐level observations. In contrast, the intra‐platform comparisons (FMO vs. SPO) are not subject to these confounders and thus provide stronger evidence for algorithmic effects. Second, although standard clinical dose constraints and commonly employed optimization weights were used, these parameters could influence the resulting dose distributions if adjusted. Consequently, the reported dose outcomes may not represent the optimal achievable results for each algorithm. In particular, manual tuning for PO introduced inherent variability that is absent in the automated FMO and SPO workflows. Third, the fixed 50‐iteration protocol may not have achieved full convergence, particularly for SPO. However, all algorithms were compared under identical iteration settings, preserving internal validity. Fourth, per‐cancer type subgroup analyses (*n* = 10) are underpowered, and the NSCLC geometric hypothesis in particular should be considered hypothesis‐generating. The study was not adequately powered to detect effect sizes below *d* = 1.02. Therefore, results with smaller effect sizes should be interpreted cautiously. These findings are exploratory and require confirmation in larger cohorts. Patient‐level data are provided as an Excel spreadsheet in the Supplementary Materials (Table S4) to allow verification of all reported results. Fifth, automated cardiac substructure delineation introduces geometric uncertainty, and even minor contour variations can alter dose metrics such as LAD dose despite manual refinement. Although all plans in this study used the same contours, cross‐study comparisons should consider differences in image quality and contouring atlases. Sixth, we did not account for the dosimetric impact of intra‐fractional motion. This represents a potential confounder, as previous breast cancer studies have shown that respiratory‐induced uncertainty disproportionately affects substructures like the LAD (mean dose variation: 0.53‐5.18 Gy) even when whole‐heart metrics remain stable.^31^ However, the specific effects of respiratory motion were not the focus of this work. By using a single static CT for each patient, our within‐subject comparison design removes respiratory‐induced variability from the comparison, ensuring that intra‐platform differences are not confounded by respiratory motion and that inter‐platform differences reflect system‐level performance, thereby preserving internal validity. Seventh, in a number of plans, one or more OAR constraints were not met before normalization, primarily due to unavoidable anatomical overlap between the PTV and the OAR, the priority given to target coverage, and relatively strict OAR dose constraints. These deviations were minor and occurred in the context of target coverage being prioritized. Because dose normalization uniformly scales all doses, OAR doses may increase proportionally when the normalization factor is greater than 1; consequently, some OARs that were marginally compliant before normalization may exceed their constraints after normalization. All algorithms underwent the identical constraint set and normalization procedure; therefore, these violations do not affect the internal validity of the intra‐platform comparison. These compliance data (Table S2) are reported transparently to allow readers to judge their clinical relevance.

Finally, this study did not include a substructure‐constrained planning arm. Therefore, the incremental clinical benefit of explicit cardiac substructure constraints over whole‐heart constraints alone remains to be quantified in future within‐platform studies.

## 5 CONCLUSION

In this dosimetric analysis, intra‐platform FMO‐versus‐SPO comparisons showed that, under the specific uRT‐TPOIS protocol studied, optimizer selection was associated with differences in cardiac substructure doses within the same TPS. Cross‐platform differences, confounded by multiple system‐level factors, revealed cancer‐specific descriptive patterns that should be interpreted as hypothesis‐generating rather than evidence of algorithmic superiority. Substructure‐based assessments uncovered dosimetric disparities that whole‐heart metrics may obscure, providing a rationale for the hypothesis that explicit cardiac substructure constraints could offer additional sparing. However, the incremental benefit of such constraints over whole‐heart constraints alone was not quantified and remains to be tested in dedicated within‐platform studies with standardized protocols and substructure‐constrained arms. Confirmatory studies with larger sample sizes are warranted to validate these exploratory findings.

## AUTHOR CONTRIBUTIONS

Chun Wu wrote the manuscript. Zhenmei Cao collected and analyzed the data. Lixiang Han, Tingting Duan, and Bin Wang designed the plans in the study. Weijie Lei designed the experiment and supervised the whole study. All coauthors have reviewed and approved the final manuscript.

## CONFLICT OF INTEREST STATEMENT

The authors declare no conflicts of interest.

## Supporting information




**Supporting Information**: acm270911‐sup‐0001‐SuppMat.docx

**Supporting Information**: acm270911‐sup‐0002‐SuppMat.tif

**Supporting Information**: acm270911‐sup‐0003‐SuppMat.docx

**Supporting Information**: acm270911‐sup‐0004‐SuppMat.xlsx

**Supporting Information**: acm270911‐sup‐0005‐SuppMat.xlsx

**Supporting Information**: acm270911‐sup‐0006‐SuppMat.xlsx

## Data Availability

The data that support the findings of this study are available from the corresponding author upon reasonable request.
