# Supplementary material for: Cardiac substructure dose in thoracic VMAT without explicit constraints: FMO versus SPO on uRT‐TPOIS with cross‐platform reference to eclipse PO
Source: J Appl Clin Med Phys. 2026 Nov 14;27(11):e70911. doi: 10.1002/acm2.70911 (PMC13769377; doi:10.1002/acm2.70911)
Supplement: Supplementary file 1 [file ACM2-27-e70911-s004.docx]

**Supplementary Table S1A**. Key dosimetric parameters at 30, 50, and 100 iterations in one randomly selected case per cancer type (esophageal, NSCLC).

| Cancer type | Algorithm/Iterations | PTV D99 | HI | CI | V105 | V107 | MHD | Spinal cord Maxdose | Lung Dmean | Lung V5 |
| --- | --- | --- | --- | --- | --- | --- | --- | --- | --- | --- |
| esophageal |  |  |  |  |  |  |  |  |  |  |
|  | FMO/30 | 4788.74 | 0.12 | 0.85 | 75.15 | 47.89 | 2149.23 | 2907.44 | 830.52 | 56.63 |
|  | FMO/50 | 4873.51 | 0.07 | 0.89 | 3.18 | 0 | 2050.54 | 2915.6 | 817.04 | 51.93 |
|  | FMO/100 | 4862.57 | 0.08 | 0.87 | 28.12 | 0.17 | 2071.43 | 2977.93 | 824.99 | 52.57 |
|  | SPO/30 | 4809.04 | 0.1 | 0.86 | 69.3 | 24.43 | 1920.16 | 3257.17 | 793.42 | 47.94 |
|  | SPO/50 | 4809.72 | 0.1 | 0.87 | 68.98 | 27.81 | 1903.52 | 3306.77 | 791.7 | 47 |
|  | SPO/100 | 4856.76 | 0.08 | 0.88 | 13.93 | 0 | 1858.9 | 3339.43 | 770.42 | 45.73 |
| NSCLC |  |  |  |  |  |  |  |  |  |  |
|  | FMO/30 | 5466.47 | 0.17 | 0.87 | 86.55 | 80.05 | 1641.03 | 4450.28 | 1405.29 | 59.93 |
|  | FMO/50 | 5679.89 | 0.11 | 0.9 | 67.87 | 27.69 | 1901.63 | 4131.68 | 1280.33 | 55.44 |
|  | FMO/100 | 5575.43 | 0.16 | 0.87 | 83.29 | 70.65 | 1919.89 | 4248.43 | 1318.99 | 56.62 |
|  | SPO/30 | 5635.83 | 0.12 | 0.87 | 77 | 47.23 | 1967.93 | 4040.07 | 1299.94 | 55.41 |
|  | SPO/50 | 5683.66 | 0.1 | 0.89 | 64.61 | 19.84 | 1891.91 | 4043.38 | 1268.97 | 55.44 |
|  | SPO/100 | 5722.17 | 0.09 | 0.9 | 46.33 | 0.94 | 1886.86 | 3996.31 | 1247.4 | 54.75 |

**Supplementary Table S1B**. Key dosimetric parameters at 30, 50, and 100 iterations in one randomly selected breast cancer case.

| Cancer type | Algorithm/Iterations | PTV4005 D99 | HI | CI | V117 | V120 | MHD | Heart V10 | Left Lung Dmean | Left lung V5 |
| --- | --- | --- | --- | --- | --- | --- | --- | --- | --- | --- |
| breast |  |  |  |  |  |  |  |  |  |  |
|  | FMO/30 | 3621.72 | 0.34 | 0.8 | 27.18 | 23.48 | 158.33 | 2.05 | 584.45 | 25.14 |
|  | FMO/50 | 3417.64 | 0.33 | 0.81 | 38.15 | 30.59 | 185.46 | 2.09 | 641.16 | 26.51 |
|  | FMO/100 | 3645.57 | 0.34 | 0.82 | 26.93 | 23.37 | 158.1 | 2.08 | 565.11 | 24.48 |
|  | SPO/30 | 3611.37 | 0.36 | 0.79 | 34.26 | 29.63 | 163 | 2.34 | 574.21 | 24.76 |
|  | SPO/50 | 3616.61 | 0.34 | 0.84 | 49.21 | 42.94 | 192.96 | 2.22 | 702.41 | 28.99 |
|  | SPO/100 | 3620.08 | 0.36 | 0.79 | 33.22 | 29.04 | 161.86 | 2.25 | 572.87 | 24.85 |
