# Supplementary material for: Cardiac substructure dose in thoracic VMAT without explicit constraints: FMO versus SPO on uRT‐TPOIS with cross‐platform reference to eclipse PO
Source: J Appl Clin Med Phys. 2026 Nov 14;27(11):e70911. doi: 10.1002/acm2.70911 (PMC13769377; doi:10.1002/acm2.70911)
Supplement: Supplementary file 2 [file ACM2-27-e70911-s006.docx]

**Supplementary Figure S1**. DVH convergence analysis for representative cases.

Each row corresponds to one cancer type (esophageal, breast, and NSCLC). DVH curves are shown for FMO and SPO plans at 30, 50, and 100 iterations. The curves illustrate changes in target coverage and organ-at-risk doses with the iteration number.
